# The paradigm shift in Antarctic ice sheet modelling

**DOI:** 10.1038/s41467-018-05003-z

**Published:** 2018-07-16

**Authors:** Frank Pattyn

**Affiliations:** Laboratoire de Glaciologie, Université libre de Bruxelles (ULB), Brussels, B-1050 Belgium

## Abstract

The Antarctic ice sheet is one of the largest potential contributors to future sea level rise. Predicting its future behaviour using physically-based ice sheet models has been a bottleneck for the past decades, but major advances are ongoing.

Unlike atmospheric models, ice sheet models emerged very recently. The first numerical Antarctic continental-scale ice sheet models, for instance, saw the light at the beginning of the 1990s. Initially, such models were employed at rather coarse resolution (∼50 km) to investigate ice sheet changes during glacial-interglacial cycles. At that time, ice sheets were believed to be a slow component of the climate system with a highly diffusive response to palaeo-climatic changes. This diffusive nature stems from the fact that ice sheet models were based on the so-called 'Shallow-Ice Approximation (SIA)', which is the dominant type of ice deformation for a large ice sheet resting on a near-frozen bed and based on the premise that ice deformation is due to shearing close to the bed. This may be valid for the bulk of ice sheet flow, but breaks down near its edges. It results in a gradual, slow response to the imposed climatology with a time lag that increases with the size of the ice sheet due to its internal thermomechanics (cold ice deforms slower than temperate ice).

Paradoxically, theoretical developments around the possibility of rapid continental change was advocated several decades before^1^, hypothesizing a possible collapse of the West-Antarctic ice sheet (WAIS) as a consequence of anthropogenic global warming^2^. The WAIS is a marine ice sheet for which the bed lies well below sea level. As the ice thins towards the edge of the ice sheet, ice thickness becomes equal to the buoyant thickness of ice and starts to float, forming ice shelves. The contact where ice starts afloat is called the grounding line. The bed is also depressed deeper in the centre of the ice sheet, making the bed slope inland from the grounding line to the centre, hence creating a reverse or retrograde bed slope. The proposed instability, known as Marine Ice Sheet Instability (MISI; Fig. 1a) is based on the observation that since ice flux increases with ice thickness, the location of a grounding line on a bed sloping inwards is unstable.

**Fig. 1:**
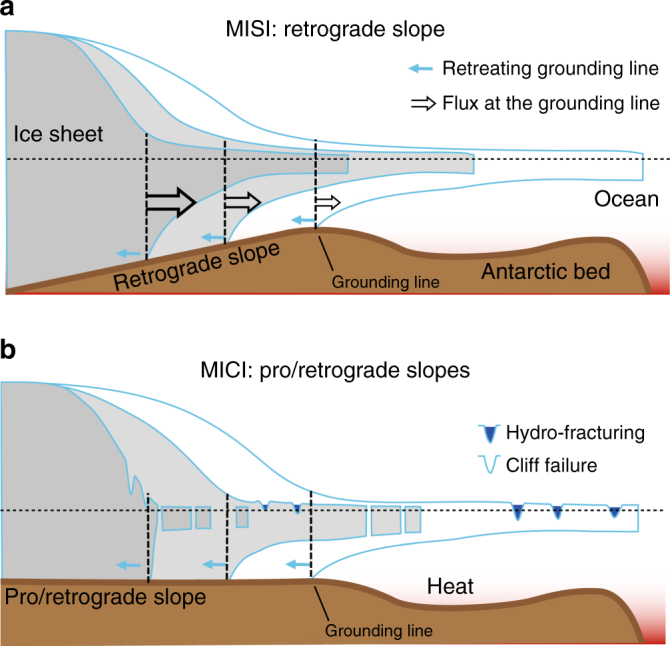
Instability scenarios. **a** Marine Ice Sheet Instability versus **b** Marine Ice Cliff Instability (MICI). Ice discharge generally increases with increasing ice thickness at the grounding line. For a bed sloping down towards the interior this may lead to unstable groundingline retreat (MISI), as increased flux (due to reduced buttressing) leads to thinning and eventually flotation, which moves the grounding line into deeper water where the ice is thicker. Thicker ice results in increased ice flux, which further thins the ice, which results in further retreat into deeper water (and thicker ice), and so on. MICI is the result of collapse of exposed ice cliffs (after the ice shelf collapses due to hydro-fracturing) under their own weight. MISI applies for a retrograde slope bed, while MICI can also apply for prograde slopes

## Floating ice matters

MISI theory was challenged by most ice sheet models, as they considered that ice shelves were too weak (and therefore mechanically uncoupled from the ice sheet) to affect the force balance of the grounded ice sheet. This led to the hypothesis of neutral equilibria that were neither stable nor unstable^3^. Not only were those SIA models unable to reproduce MISI, the theory was also disputed by observations of an apparently balanced ice sheet, but that was before glaciers started to retreat in the Amundsen Sea Embayment (ASE) of the WAIS^4^.

Mathematical proof of the MISI theory put forward by Weertman was finally given three decades later^5^, dispelling the previous idea that ice sheets and ice shelves are mechanically uncoupled. This had a profound impact on ice sheet model development, pushing models to conform to known (analytical) solutions, which led to international model intercomparisons, such as the Marine Ice Sheet Model Intercomparison Project (MISMIP)^6^. MISMIP allowed for a collective improvement of marine ice sheet models by adapting both their physical basis as well as their numerical approaches. Besides being the limit of flotation, a grounding line is also the change from a shear-dominated ice flow to an ice flow dominated by longitudinal pushes and pulls (due to membrane stresses). The transition is never sharp, but gradual, and largely depends on the processes that govern basal motion under ice sheets within fast-flowing ice streams (basal sliding and sediment deformation). These membrane stresses need to be resolved across the grounding line with a sufficiently high spatial resolution, which pushed the development of spatial grid refinement in numerical models through sub-grid interpolation schemes, the use of unconstructed grids or adaptive mesh approaches^7^. It also requires the use of approximations other than the SIA to the flow of ice, ranging from the Shallow-Shelf Approximation to full-Stokes models, which are harder to solve.

Despite these theoretical and numerical advances, the developed MISI theory^5^ remains only valid for unconfined ice shelves, i.e. ice shelves that do not exert a force on the inland ice sheet other than the (ocean) water pressure. In reality, however, ice shelves are bounded within embayments, thereby exerting a back force to the grounding line, hence limiting the flow speed of ice streams and their discharge of ice through the ice shelf into the ocean. This is commonly known as the buttressing effect of ice shelves that act as a cork on a bottle, preventing the ice to flow out too fast. Loss of ice-shelf buttressing has effectively been witnessed in Antarctica at the beginning of the 21st century, where ice shelf collapse in the Antarctic Peninsula led to increased glacier discharge^8^.

Ice shelf weakening happens through both atmospheric and ocean processes, such as hydro-fracturing and sub-shelf melting. Hydro-fracturing is a process that increases the water pressure from surface melt on ice shelves in surface crevasses, thereby widening them so that they become more vulnerable to calving^9^. This eventually leads to ice shelf collapse if sufficient melt water and cracks are available. Hydro-fracturing is also considered a precursor for a new emerging mechanism in ice sheet modelling, the concept of Marine Ice Cliff Instability (MICI; Fig. 1b), i.e., that once ice shelves have collapsed ice cliffs become unstable and fall down if higher than ∼90 m above sea level, leading to accelerated collapse of ice sheets^10^. MICI is a process that facilitates and enhances MISI and relies on the assumption of perfect plastic rheology to represent failure. However, these crucial processes of ice shelf breakup (hydro-fracturing) and calving front/cliff stability still need to be further explored. While such mechanisms aid at explaining past changes in the Antarctic ice sheet, they do show a higher sensitivity to forcing, and hence lead to a significant larger mass loss^10^.

Sub-shelf melting is responsible for more than half of the ice mass loss at the margins of the Antarctic ice sheet^11^. As with calving due to hydro-fracturing, sub-shelf melting decreases the buttressing capacity of ice shelves via loss of pinning points and weakening of the ice shelf through thinning. This has presumably been the trigger of the observed acceleration of large Antarctic outlet glaciers in the ASE during the last decade. However, sub-shelf melt is particularly determined by ocean circulation within the ice shelf cavity, which, in turn, requires high-resolution ocean circulation models to link large-scale ocean circulation to sub-shelf melt. While such ongoing and future developments require increased computer power, intermediate solutions can be obtained through physically based parametrisations of sub-shelf melt based on sub-shelf ocean circulation^12^.

## Towards decadal predictability of ice sheets?

A key aspect of projecting future Antarctic mass loss with dynamical ice sheet models is related to the initial state of the model. Since ice sheet models were initially applied for palaeo-climatic studies on long time scales, initialisation was generally obtained from a long spin-up time leading to a steady-state ice sheet (both in terms of geometry and thermodynamics). However, for predictions on shorter time scales (decades to centuries), a stable spin-up generally leads to an ice sheet geometry far different from the one currently observed, which is one of the reasons why such ice sheet models may respond differently than observations suggest^13^. Moreover, using a steady-state for initialising the ice sheet prevents models from properly accounting for the dynamical mass losses observed over the last decade, as the present-day ice sheet is not in steady state. Motivated by the increasing ice sheet imbalance of the ASE glaciers over the last 20 years, and supported by the recent boom in satellite data availability, data-assimilation methods are progressively used to evaluate unknown fields using time-evolving states accounting for the transient nature of observations and the model dynamics^14^.

Antarctic ice sheet modelling has taken a big step forward based on an improved understanding of key processes and the development of assimilation methods leading to the ability to reproduce observed ice sheet changes and by making refined future projections. From a theoretical viewpoint, we now have the ability to verify marine ice sheet models, albeit that certain effects, such as buttressing, are not yet quantified/verified accurately, but new analytical tools are becoming available^15^. The increase in computational efficiency enabling high spatial resolution modelling, high-resolution datasets of bedrock topography and surface velocity, longer time series on ice sheet changes, and the improved initialisation of ice sheet models are now allowing ice sheet modelling to move away from the slow-diffusive response over millennium time scales towards robust predictions on decadal time scales, hindcasting and potentially reanalysis.
